# Supplementary figures and images for: Lipopolysaccharide induces mouse translocator protein (18 kDa) expression via the AP-1 complex in the microglial cell line, BV-2
Source: PLoS One. 2019 Sep 19;14(9):e0222861. doi: 10.1371/journal.pone.0222861 (PMC6752844; doi:10.1371/journal.pone.0222861)

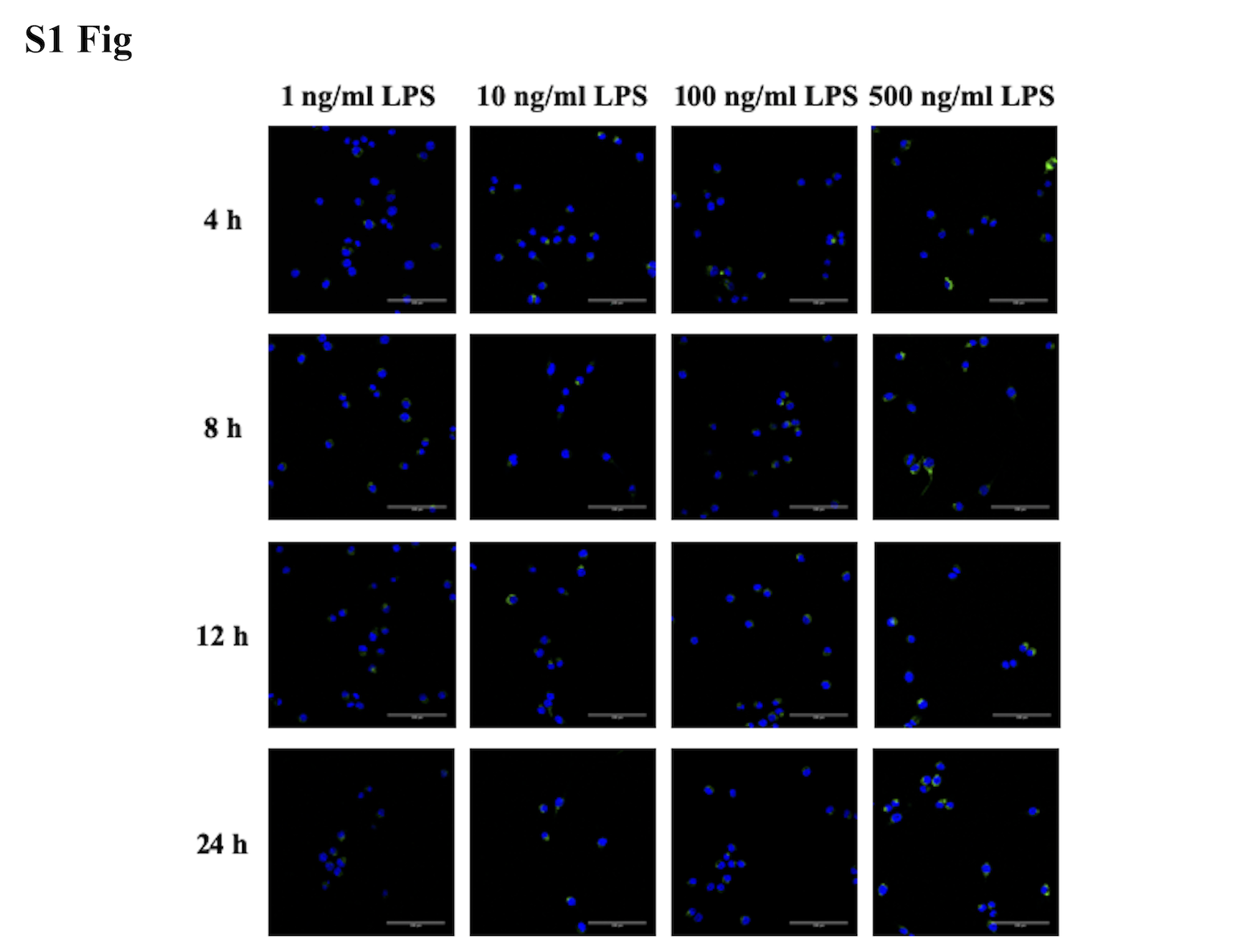

Supplement: S1 Fig — BV-2 cells were stimulated with 1, 10, 100 or 500 ng/ml LPS for 4, 8, 12 or 24 hours and immunocytochemistry was then carried out. Images of nuclear (DAPI, blue) and TSPO (green) staining were acquired at 40× magnification. Scale bar: 100 μm. (TIFF) [file pone.0222861.s003.tiff]

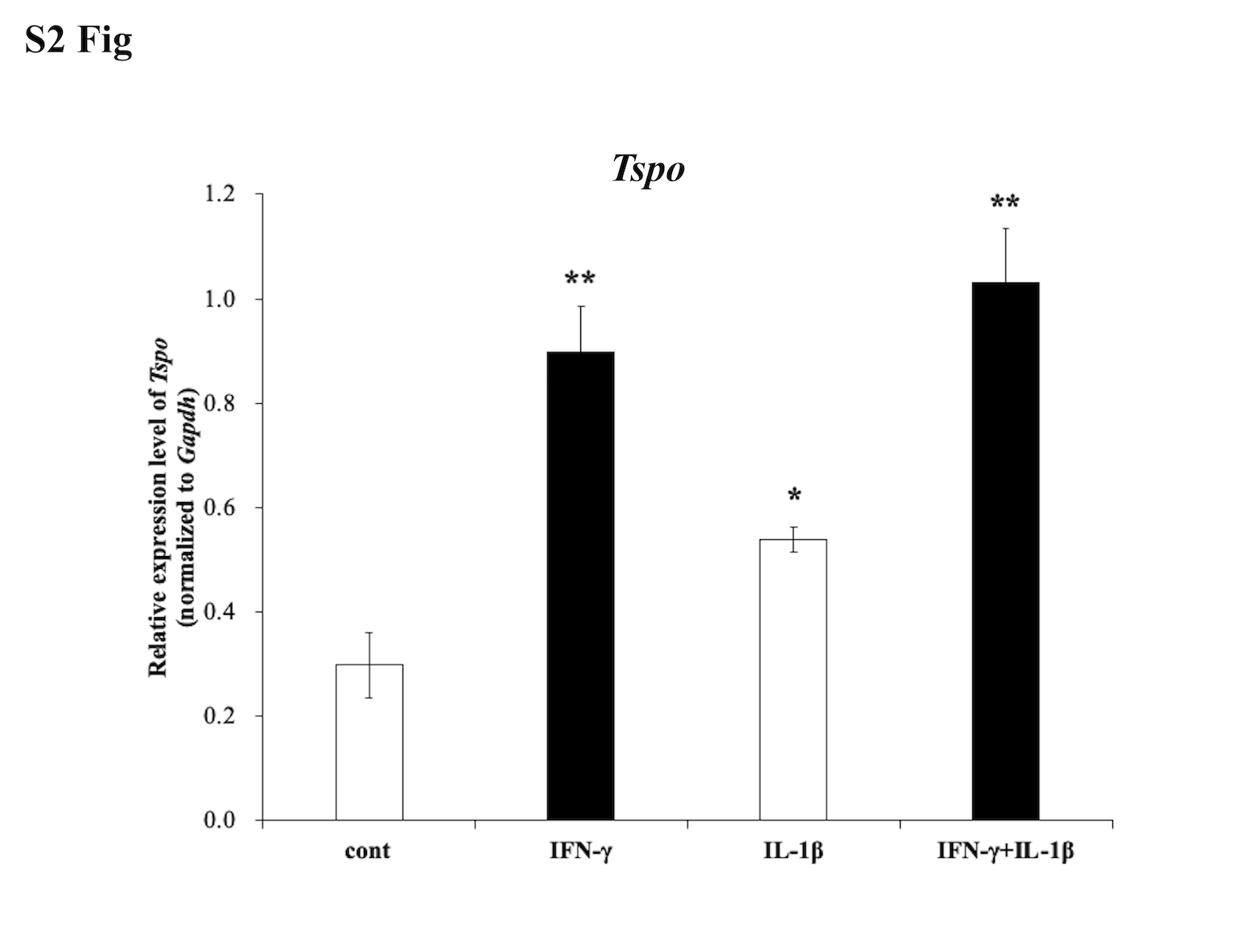

Supplement: S2 Fig — BV-2 cells were treated with 100 ng/ml IFN-γ and/or IL-1β for 8 hours and RNA was then extracted and subjected to qRT-PCR. The mRNA levels were measured and normalized to those of Gapdh mRNA. The asterisks indicate statistically significant differences compared to the control (n = 3, *p<0.05, **p<0.01). (TIFF) [file pone.0222861.s004.tiff]
