# Supplementary material for: Lipopolysaccharide induces mouse translocator protein (18 kDa) expression via the AP-1 complex in the microglial cell line, BV-2
Source: PLoS One. 2019 Sep 19;14(9):e0222861. doi: 10.1371/journal.pone.0222861 (PMC6752844; doi:10.1371/journal.pone.0222861)
Supplement: Attachment — Submitted filename: Response to Reviewers.docx [file pone.0222861.s005.docx]

**Response to editor**

2. We note that you have included the phrase “data not shown” in your manuscript. Unfortunately, this does not meet our data sharing requirements. PLOS does not permit references to inaccessible data. We require that authors provide all relevant data within the paper, Supporting Information files, or in an acceptable, public repository. Please add a citation to support this phrase or upload the data that corresponds with these findings to a stable repository (such as Figshare or Dryad) and provide and URLs, DOIs, or accession numbers that may be used to access these data. Or, if the data are not a core part of the research being presented in your study, we ask that you remove the phrase that refers to these data.

**Response:**

**In discussion section, we mentioned the subcellular localization of TSPO based on our previous version of immunocytochemical data and described as “data not shown” because of the obscure results. To meet the PLOS One’s publication criteria, we examined and provide new immunocytochemical data into Fig.2C. We revised sentence according to the additional results.**

**Another point of “data not shown”, we deleted following sentence because it is not pivotal for this study.**

**“In our preliminary experiments (data not shown), expression of the TSPO protein was altered in the cerebellum, ACC and striatum in Fmr-1 KO mice, which is an ASD model. “**

**Response to Reviewer**

Reviewer #1: Comments to authors:

In this study, Shuji Shimoyama et al. have demonstrated evidence that lipopolysaccharide (LPS) induced Tspo expression which activated by the AP-1 complex in mouse microglia cell line, BV-2. Basically they investigated that knockdown of c-Fos, a component of AP-1, reduced LPS-induced Tspo expression. Moreover, enrichments of Sp1 in the proximal promotor region of Tspo were increased in the presence of LPS. This research article has presented novel finding in regards to 18-kDa translocator protein TSPO as a target to image neuroinflammation as its expression in upregulated in reactive glial cells during CNS pathologies. This manuscript could be strengthen by applying following comments.

Major revision:

1. In the results section, the authors examined the high and low concentrations of LPS treatment induced Tspo expression in BV-2 cells. It would be required to perform microscopic images of different time point and concentrations.

**Response:**

**Thank you for your advice. We performed immunocytochemical analysis of TSPO expression in LPS-treated BV-2 cells. BV-2 cells were stimulated with 1, 10, 100 or 500 ng/ml LPS for 4, 8, 12, 24 h and then stained with anti-TSPO antibody (green) and DAPI (blue). As well as the results of qRT-PCR and Western blotting analysis, the fluorescent signal for TSPO was increased at high concentration of LPS. We depict the results of 8 h LPS treated group inFig.1E as representative data, and other results were shown in S2 Fig.**

2. In regards to AP-1 components (c-Fos and c-Jun), the authors have performed the knockdown of c-Fos reduced Tspo expression. To strengthen the data I recommend to perform the knockdown experiment of c-Jun component. Also immunofluorescence data will present more information.

**Response:**

**Thank you for the constructive comments to improve our study. According to the suggestion, we performed knockdown experiment of c-Jun using siRNA and analyzed expression of TSPO by various methods including immunocytochemistry (Fig.5). The knockdown of c-Jun reduced LPS-induced *Tspo* expression as shown in Fig.5. These results indicated that not only c-Fos but also c-Jun were essential for LPS-induced transcriptional activation of *Tspo*. Therefore, the involvement of AP-1 complex in *Tspo* transcriptional activation induced by LPS stimulation was strengthen.**

3. Authors have used lipopolysaccarid LPS as the only pro inflammatory agent to induce Tspo expression. To prove the mechanism of Tpso expression it would be needed to apply other pro inflammatory cytokines such as interferon gamma (IFNy), interleukin (IL)-1β, or a combination of them.

**Response:**

**Thank you for your comments. We examined whether IFN-γ and /or IL-1β induce transcriptional activation of *Tspo*. As shown in S2 Fig., IL-1β, IFN-γ, or both treatment increase *Tspo* expression as well as LPS treatment.**

**IFN-γ activates JAK-STAT pathway and STAT acts as transcriptional activator of inflammatory genes. The consensus sequence of STAT3 is located in the upstream of mouse *Tspo* gene overlapping with AP-1 and Ets sites (Reference #25), and hence the expression of *Tspo* was induced by IFN-γ stimulation.**

**IL-1β signaling pathway activates transcription factors via MAPKs, such as p38 MAPK, JNK and ERK. (Reference #35). Similar to LPS stimulation, IL-1β stimulation induced the expression of *Tspo* by AP-1 activation via those signaling pathways.**

4. In Fig 2 it is not clear that the data was obtained in which time point of LPS treatment.

**Response:**

**We are sorry not to have explained sufficiently in Results section. The detailed information was described only in figure legend. We added the sentence as follows in Results section. “The BV-2 cells were collected and prepared cell fraction for Western blotting after stimulating with 100 ng/ml LPS for 8 h.”**

Minor point:

1. The manuscript has not been supported by enough explanation and details for cell line construction, cell seeding and characterization.

**Response:**

**BV-2 cells were established Blasi E. *et al* (*J Neuroimmunol,* 1990, May;27 (2-3):229-37, PMID:2110186) and widely used as murine microglial cell line. We added information about cell number for each experiment.**

Reviewer #2: The manuscript by Shimoyama et al. describes a mechanism of transcriptional regulation of Tspo expression during LPS treatment of immortalized BV2 microglial cells. The authors use a variety of approaches including ChIP and siRNA knockdown to show the role of AP-1 and HDAC1 in LPS-induced Tspo expression. The data for the most part support these conclusions; however, there are several issues that should be addressed:

1) A major limitation of the study is the sole use of the BV2 cell line. As the authors state in the discussion section, the expression of Tspo is differentially regulated during lifetime. Based on the developmental time point associated with the origin of BV2 cells, there is a possibility that epigenetic and regulatory factors could be different compared to primary microglia that are relevant to functional outcomes in the context of normal CNS function as well as neuroinflammatory conditions. Validation of some aspect of the proposed mechanism in primary cells and/or an in vivo neuroinflammatory model would strengthen the study.

**Response:**

**Thank you for your constructive comments to improve this study. As you pointed out, we also wondered the transcriptional regulation of *Tspo* in primary microglial cells or *in vivo*. However, it is difficult to perform the experiment to identify which transcriptional factors are involved in LPS-stimulation because of the low amount of the primary microglial cells from adult mouse (approximately 2.0~3.0×10^4^ cells/individual, Reference #20). Therefore, we only examined the *Tspo* mRNA expression by qRT-PCR in the presence or absence of LPS.** **As shown in Fig.1B, *Tspo* expression was up-regulated in the presence of LPS in primary microglial cells from adult mouse.**

2) The authors investigate the regulatory mechanism of Tspo expression through LPS stimulation. It is not clear if TLR-mediated induction of pro-inflammatory response is relevant to the neuroinflammatory conditions mentioned in the manuscript (e.g., chronic pain, AD, etc.). Investigation of additional inflammatory mediators or at least more rationale to support this sole treatment is necessary. Additionally, given the possibility of Tspo to play a role as an anti-inflammatory factor, further investigation of regulation of Tspo expression in a broader range of activation phenotypes would strengthen study.

**Response:**

**Thank you for your constructive comments. We should have mentioned about the point in discussion section or examined about another inflammatory mediator for transcriptional activation of *Tspo*. According to reviewer’s suggestion, we examined whether IFN-γ and /or IL-1β induce transcriptional activation of *Tspo*. As shown in S2 Fig., IFN-γ, IL-1β or both treatment increase *Tspo* expression as well as LPS treatment.**

3) In order to confirm LPS-induced activation of BV2 cells, quantitation of other markers should be presented in addition to Tspo (e.g., iNOS, pro-inflammatory cytokines, etc.).

**Response:**

**We assessed the expression of *Tnf-α*, *Il-1β* and *Il-6* as pro-inflammatory markers. As reported previously, LPS stimulation induced the expression of these genes (Fig.1A).**

4) The main approach for protein detection and quantitation was antibody-based. Given the issue with specificity associated with detection, further information regarding authentication of antibodies used in this study should be reported. The authors list the company and item number for each antibody, but criteria used for in-lab authentication (e.g., positive and negative controls) were not reported.

**Response:**

**Antibodies used in this study were widely used at many publications according to manufacturer’s information (cited by 34 publications). Therefore, we did not examine the specificity of antibodies using peptide absorption, or recombinant protein as positive control and knockdown experiment as negative control.**

**In western blotting analysis, we estimated the molecular weight from the mobility and concluded that it was the target molecule. MagicMark^TM^ XP Western Protein Standard (ThermoFisher Scientific, #LC5602) and Precision Plus Protein Dual Color Standards (Bio-Rad, #1610374) were used for the measurement of mobility and estimation of molecular weight.**
